# Cadmium, road dust and rheumatoid arthritis: an alternative hypothesis to general air pollution

**DOI:** 10.1186/s12950-015-0103-2

**Published:** 2015-10-12

**Authors:** Daniel Murphy, David Hutchinson

**Affiliations:** Rheumatology Department, Royal Cornwall Hospital, Truro, Cornwall UK

**Keywords:** Rheumatoid arthritis, Dust, Cadmium, Air pollution, Citrullination

## To the Editor

We read the review “Is air pollution a risk factor for rheumatoid arthritis?” with great interest [1]. We agree that air pollution is a potential risk factor for rheumatoid arthritis (RA) in low-and-middle-income countries. As the authors correctly point out, epidemiological studies in high-income countries which have failed to demonstrate a link between air pollution and the risk of RA [2, 3], can not necessarily be generalised to individuals living in low-and-middle-income countries.

However, it remains that two large well conducted studies in North America have clearly demonstrated an increased risk of RA in those residing ≤ 50 m from a highway compared with residence > 150 m away [4, 5]. This finding is not explained by industrial pollution per se as two studies in high income countries found no consistent association between air pollution and an increased RA risk [2, 3]. If air pollution in high-income countries is not associated with the development of RA, what other factor can explain the risk for RA living in close proximity to a main road as opposed to just 150–200 m further away? We suggest a novel hypothesis of cadmium-containing road dust inhalation either by the roadside or within the home as a plausible explanation.

The lung is now considered to be an important initiating site of seropositive RA as a result of local anti-citrullinated peptide (anti-CCP) antibody generation [6]. Inhalation of cadmium has been hypothesised as a potential trigger for RA as cadmium links smoking, the most important etiological factor in the development of seropositive RA, and many of the other known contemporary risk factors such as low socio-economic class, regional clustering in the U.S. and specific occupations associated with RA [7]. Increased cadmium exposure in an animal model has recently been demonstrated to enhance disease activity of collagen induced arthritis [8].

Cadmium levels have been shown to markedly decrease within 20 m from the roadside [9]. Cadmium is a component of petrol and diesel fuel and was previously used as a curing agent in tires, a component of brake pads, alloyed with copper in the production of car radiators and in car paints. Additionally, asphalt concrete road surfacing contains appreciable amounts of cadmium [10]. We suggest that cadmium-laden ultrafine dust will occur in close proximity to busy roads as a result of both vehicular component and road surface wear, and vehicular fuel emissions. This would be particularly evident on fast moving roads where acceleration and hard braking is commonplace.

A number of nanoparticles, including silica, can induce lung citrullination via activation of cellular calcium channels with a subsequent rise in intracellular calcium and activation of peptidyl arginine deiminase and subsequent peptide citrullination [11]. Fine cadmium dusts have the potential to cause citrullination as cadmium is a potent activator of calcium channels and significantly raises intracellular calcium levels [12, 13].

Cadmium levels need not correlate with air pollution per se. This is exemplified by a US study demonstrating the mean concentration of cadmium in the air of 28 US cities was found to correlate with mortality rates from hypertension and coronary heart disease although indices of air pollution did not correlate with mortality from hypertension and coronary heart disease [14].

We suggest that further studies investigating the link between residing close to a main road and RA development take into account bodily cadmium levels as a co-founding factor as it is known that inhalation of road dust containing cadmium correlates with increased bodily levels of cadmium [15].
